# Should Pneumococcal Serotype 3 Be Included in Serotype-Specific Immunoassays?

**DOI:** 10.3390/vaccines7010004

**Published:** 2019-01-03

**Authors:** Ezra Linley, Abigail Bell, Jenna F. Gritzfeld, Ray Borrow

**Affiliations:** 1 Vaccine Evaluation Unit, Public Health England, Manchester Royal Infirmary, Oxford Road, Manchester M13 9WL, UK; Abigail.Bell@phe.gov.uk (A.B.); Ray.Borrow@phe.gov.uk (R.B.); 2 UK Experimental Arthritis Treatment Centre for Children, Department of Women’s and Children’s Health, Institute of Translational Medicine (Child Health), University of Liverpool, Institute in the Park, Alder Hey Children’s NHS Foundation Trust Hospital, Eaton Road, Liverpool L12 2AP, UK; J.Gritzfeld@liverpool.ac.uk

**Keywords:** *Streptococcus pneumoniae*, serotype 3, conjugate vaccine, multiplex, immunoassay

## Abstract

Since the introduction of the 13-valent pneumococcal conjugate vaccine, a number of studies have demonstrated the limited efficacy of the pneumococcal serotype 3 component of this vaccine. Evidence from seven countries (Denmark, France, Greece, Portugal, Sweden, UK, US) shows limited or no effectiveness of the 13-valent pneumococcal conjugate vaccine against serotype 3 invasive pneumococcal disease and carriage. The serotype 3 capsule has some unique characteristics that may serve to explain this lack of efficacy—capsular polysaccharide is abundantly expressed, leading to a greater thickness of capsule, and free capsular polysaccharide may be released during growth. The serotype 3 component of the Luminex multiplex assay demonstrates inferior inter-laboratory reproducibility than other components and results may not be reliable. This communication outlines this evidence and discusses whether it is necessary to include serotype 3 in the assay in the future.

## 1. Introduction

*Streptococcus pneumoniae* is endemic throughout the world and is a significant cause of mortality and morbidity. The introduction of polysaccharide conjugate vaccines (PCVs), starting with the seven-valent PCV7 in the early 2000s and the 13-valent PCV13 from 2010 onwards has seen a substantial decrease in the incidence of invasive pneumococcal disease (IPD) [1].

Of the 13 serotypes in PCV13 (1, 3, 4, 5, 6A, 6B, 7F, 9V, 14, 18C, 19A, 19F and 23F), serotype 3 is of interest because, whilst it is relatively rare in childhood IPD (estimated as 4.9% of cases globally [2]), it is one of the predominant causes of paediatric complicated pneumococcal pneumonia (PCPP) [3] and otitis media (OM) [4], and is significantly more common in IPD in older children and adults [5].

Measures of serum antibody levels are useful for population vaccine efficacy studies, and for clinical investigations to determine vaccine efficacy in individuals; pneumococcal vaccines are a commonly-used tool to evaluate humoral immune response [6]. In order to overcome the expense and difficulty of running multiple enzyme-linked immunosorbent assays (ELISAs) to evaluate antibody levels, multiplex immunoassays have been developed, the first of which utilised the Luminex platform [7]. Such immunoassays are now in common use in multiple laboratories.

Since the introduction of PCV13, a number of studies have demonstrated the limited efficacy of the serotype 3 component of this vaccine [8,9,10,11,12,13,14,15,16,17,18]. A previously developed 11-valent conjugate vaccine (PCV11-PD) [19], and the 23-valent polysaccharide vaccine (PPV23) [20], have also demonstrated limited efficacy. This communication outlines the evidence for this limited efficacy, gives reasons why serotype 3 protection from polysaccharide-conjugate vaccines may be difficult, and illustrates a technical issue with the serotype 3 component of the multiplexed pneumococcal antibody assay. We go on to discuss the limited utility of a measurement of serotype 3-specific antibodies and question whether it is necessary to include serotype 3 in the assay in the future.

## 2. PCV13 Efficacy against Serotype 3

### 2.1. Efficacy against Invasive Pneumococcal Disease

PCV13 was introduced into the Danish schedule in 2010 and coverage for all three doses of the vaccine is estimated to be 80% at 12 months. Since 2007, it has been mandatory for all clinical microbiology laboratories in Denmark to submit all IPD-causing isolates to the Statens Serum Institute (SSI) for serotype identification. Using data from the SSI, Slotved et al. [8] analysed the incidence of serotype 3 IPD from 1999 to 2016 to compare incidence before and after the introduction of the vaccine. They found no significant difference in serotype 3 IPD incidence between the periods pre- and post-vaccine in any of the three age groups investigated. They concluded that PCV13 was not providing herd protection from serotype 3, but could suggest no mechanism for this.

PCV13 was introduced into Portugal in 2010 though neither it, nor PCV7, were part of the national immunisation programme until 2015. The Portuguese Group for the Study of Streptococcal Infections (PGSSI) has monitored IPD since 1999, and 31 laboratories provide isolates from IPD cases for serotyping, though this is not mandatory. Using data from the PGSSI, Horácio et al. [9] analysed the incidence of IPD in adult patients caused by all serotypes from 2012 to 2014. They found that, whilst the majority of serotypes in PCV13 showed a decrease during this time, serotype 3 remained unchanged. A second study of IPD cases in paediatric patients in Portugal between 2010–2015 found evidence of serotype 3 infection in 17 cases of children appropriately vaccinated with PCV13 [10]. The authors found that serotype 3 prevalence was greater among children having completed the PCV13 course than among other patients of known vaccination status. 

PCV13 was introduced into the US schedule in 2010. The Active Bacterial Core surveillance (ABCs) service of the Centers for Disease Control (CDC) collects isolates from IPD cases across ten sites around the U.S. Moore et al. [11] used the service to identify IPD cases and perform serotyping on isolates collected between 1st July 2004 and 30th June 2013. A total of 9830 isolates from the period following the introduction of PCV13 were serotyped (estimated mean PCV13 coverage was 76% for eligible children during this period). The authors found a 64% reduction in the incidence of IPD cases caused by all PCV13/non-PCV7 serotypes; however examination of individual serotypes found no evidence for a reduction in serotype 3, and the authors concluded that the effectiveness of PCV against serotype 3 required further study.

PCV13 was introduced into the UK schedule from 1st April 2010. Using enhanced surveillance data from Public Health England (PHE), Andrews et al. [12] carried out an indirect cohort study of the effectiveness of PCV13 against the individual serotypes covered by the vaccine. All cases of IPD in England and Wales in the cohort eligible for the PCV13 vaccine between 1st April 2010 and 31st October 2013 were analysed, and vaccine effectiveness was estimated using a case-control design whereby the cases were individuals with vaccine-type IPD and controls were individuals with IPD caused by non-PCV13 serotypes. The authors estimated the effectiveness of PCV13 as 79% against the serotypes not covered by PCV7, however the estimated effectiveness against serotype 3 was 26%, with a 95% confidence interval of −69 to 68% (i.e., covering zero). As part of this study, Andrews et al. also sought to obtain a more suitable correlate of protection value than the previously accepted value of 0.35 μg/mL, using a combination of ELISA to measure antibodies against PCV13 serotypes, combined with opsonophagocytic assay of a random subset of 100 samples. The authors concluded that 0.35 μg/mL is an under-estimate, and provided a revised value of 2.83 μg/mL for serotype 3. A second study conducted by Oligbu et al. [13] of IPD cases in children aged less than 5 years occurring between 4th September 2006 and 2nd September 2014 identified 28 cases of vaccine failure in children receiving a full course of PCV13. Of these, 12 (43%) were serotype 3 infections. The authors concluded that vaccine failure was more common for serotype 3.

Following the introduction of PCV7 into the Swedish schedule in 2007, there was an unexpected increase in the rate of infection due to serotype 3 [14]. PCV13 was introduced into the Swedish schedule in 2010, and coverage is currently estimated at 97%. It is mandatory to report all cases of IPD in Sweden to the Public Health Agency of Sweden, and isolates are taken from all cases. Galanis et al. [14] used these isolates to study the incidence of IPD in Stockholm County from 2005 to 2014. Serotyping data were used to compare the period prior to the introduction of the PCV7 vaccine, post-PCV7 introduction and post-PCV13 introduction. They found an 18% decrease in IPD incidence in the entire population following childhood PCV7 introduction, and a further 11% reduction following childhood PCV13 introduction, however there was no reduction in IPD due to serotype 3.

In Spain, PCV13 was licensed from 2010 but not financed by the Catalan Public Health System until 2016, and not introduced into the Spanish recommended calendar until January 2017. Consequently, coverage in Spain was low (estimated as 55% in Catalonia between 2012–2013), nevertheless several vaccine failures against IPD caused serotype 3 had been observed by 2016 [15]. A matched case-control study by Domínguez et al. involving 814 children aged 7–59 months (169 IPD cases and 645 controls) was carried out in three hospitals in Barcelona between January 2012 and June 2016. They found a non-significant vaccine effectiveness against serotype 3 of 25.9% (95% CI, −65.3% to 66.8%), and concluded that the vaccine is not effective against this serotype [15]. 

It should be noted that not all studies of PCV13 efficacy have concluded that the vaccine is not efficacious against IPD due to serotype 3. In a matched case-control study in the US described by Moore et al. [21] involving 3713 children aged 2–59 months (722 with IPD and 2991 controls) between 01/05/2010 and 31/05/2014, effectiveness of at least one dose of PCV13 against serotype 3 IPD was found to be 79.5% (95% CI, 30.3% to 94.8%). In an indirect cohort study in Germany between July 2006 and June 2015, van der Linden et al. [22] indentified 618 IPD cases in children aged between 74–729 days for which vaccination status could be established and estimated PCV13 effectiveness against serotype 3 as 74% (95% CI, 2% to 93%). 

A commentary by De Wals [23] aimed to explain these apparently paradoxical findings whereby some studies find positive effectiveness of PCV13 against serotype 3, whilst the majority found no evidence of effectiveness. De Wals suggests that the protection against serotype 3 afforded by PCV13 might be of short duration, citing evidence of a case-control study from Quebec estimating effectiveness of 79% (95% CI, −148% to 98%) in the first 365 days following the vaccination course, and −199% (95% CI, −2004% to 98%) more than 365 days after the last dose [24], along with evidence from the Andrews et al. UK study [12], which found effectiveness of 66% (95% CI, −322% to 92%) amongst children observed between 4 and 11 months of age, decreasing to 26% (95% CI, −69% to 68%) amongst children between 4 and 55 months of age. He also suggests that a single toddler dose of PCV13 may be more effective than a 2 + 1 or 3 + 1 schedule, due to a “phenomenon of immunotolerance after repeated vaccinations” citing evidence from a number of studies showing lower antibody levels after booster doses of conjugate vaccines against serotype 3 than immediately after the primary dose [19] or lower antibody levels in children receiving 2 + 1 or 3 + 1 doses of PCV13 than those receiving primer doses of non-serotype 3 containing conjugate vaccines followed by a single dose of PCV13 [25,26]. 

### 2.2. Efficacy against Otitis Media

PCV13 was introduced into the French schedule in 2010. Cohen et al. [16] carried out a study to investigate the effect of the PCV13 programme on pneumococcal carriage in infants diagnosed with acute otitis media (AOM) between October 2010 and May 2011 at 58 surgeries throughout France. The authors found that overall carriage of the six PCV13-specific serotypes (i.e. those present in PCV13 but not in PCV7) was reduced by 49.2% following the introduction of PCV13, but that there was no significant effect on serotype 3 carriage alone. A further study by Cohen et al. [17] covering the 13 years from October 2001 to June 2014 and involving 121 paediatricians throughout France again showed no significant effect of the PCV13 programme on serotype 3 carriage associated with AOM.

A study by Lewnard et al. [27] aimed to investigate the efficacy of PCV7 and PCV13 introduction at reducing the rate of progression from carriage to OM of various pneumococcal serotypes in children in Israel. They analyzed data from previously-published studies of pneumococcal carriage and OM incidence among Bedouin and Jewish children in the Negev region of southern Israel and determined progression rate as the rate of OM incidence divided by carriage prevalence for each serotype. They concluded that there was a significant reduction in progression to OM due to serogroup 3, however it should be noted that similar reductions were seen in non-vaccine serotypes and, in the study from which the OM incidence data were taken (Ben-Shimol et al. [28]), a similar reduction was also seen in non-pneumococcal OM, which the authors attribute to prevention of early pneumococcal OM episodes. Consequently, it appears impossible to determine whether reductions are due to direct effects of vaccination. 

### 2.3. Efficacy against Carriage

Dagan et al. [18] carried out a randomised, double-blind trial aimed at comparing the efficacy of PCV7 versus PCV13 in reducing nasopharyngeal (NP) colonisation in infants in Israel. Healthy infants were randomised to receive either PCV7 or PCV13 at 2, 4, 6, and 12 months of age. NP swabs were taken at 2, 4, 6, 7, 12, 13, 18 and 24 months of age and blood samples were taken at 7 and 13 months of age. Rates of NP colonisation were assessed using culture, isolates were serotyped using the Quellung reaction, and IgG levels were measured using ELISA. Of 801 patients receiving PCV13, 16 were colonised by serotype 3 after 24 months and of 804 patients receiving PCV7, 16 were colonised by serotype 3. IgG GMCs were 0.97 μg/mL for the PCV13 group and 0.04 μg/mL for the PCV7 group. The authors concluded that there was no impact on colonization from the PCV13 vaccine.

## 3. Efficacy of Other Vaccines against Serotype 3

Following the introduction of PCV7, an 11-valent vaccine consisting of 11 pneumococcal polysaccharides conjugated to recombinant non-lipidated *Haemophilus influenzae* protein D (PCV11-PD) was developed. Prymula et al. [19] conducted a randomised double-blind trial to test the efficacy of PCV11-PD against AOM in infants in the Czech Republic and Slovakia. 4968 infants were randomly assigned to receive either PCV11-PD or hepatitis A vaccine at 3, 4, 5, and 12–15 months and were followed-up until the end of the second year of life. Samples of middle ear fluid were taken for culture and serotyping from children presenting with symptoms of AOM. The group receiving PCV11-PD had a significant (57.6%) reduction in AOM incidence from all vaccine serotypes; however efficacy against serotype 3 was estimated at −17.1% (i.e., no efficacy). Subsequently, serotype 3 was removed from the 11-valent vaccine, which became the 10-valent PCV10.

PPV23 includes serotype 3 polysaccharide in its formulation and was introduced into the UK schedule for every person over 65 in 2003. Andrews et al. [20] analysed data from IPD enhanced surveillance in England and Wales between 1998–2010, and estimated PPV23 effectiveness against serotype 3 as −23% (i.e., not effective), the lowest of the 23 serotypes covered by the vaccine (effectiveness ranged from −12 to 63% for the other 22 serotypes).

## 4. Reasons for Lack of Vaccine Efficacy

Poolman et al. [29] sought to investigate the reasons for the lack of efficacy of PCV11-PD against serotype 3 by culturing colonies of a number of serotype 3 strains and examining the phenotypes of the resulting colonies, including electron microscopy (EM) of the capsule. The authors found that serotype 3 strains produced mucoid colonies (with the exception of the small colony forming strain SSI 3/1), in contrast to the other 10 serotypes included in PCV11-PD. EM images displayed very dense capsules of about 200 nm thickness, with the exception of SSI 3/1 which had a “somewhat thinner and strikingly less dense” capsule. Opsonophagocytic assays demonstrated low activity against the densely capsulated serotype 3 strains compared to other vaccine serotypes and SSI 3/1. It has previously been shown that the ability of antibodies to kill a pneumococcal strain by opsonophagocytosis is reduced as the thickness and/or density of the polysaccharide capsule increases [30]. Poolman et al. suggested that this atypical capsule may make serotype 3 less susceptible to anti-polysaccharide antibody-mediated defence mechanisms. They also discussed evidence that down-regulation of capsule production is an important virulence factor for serotype 3, and speculated that this may be due to large capsules preventing serotype 3 from associating with biofilms. Evidence discussed suggests the importance of biofilms for the progression of AOM, and that serotype 3 grown in biofilms in vitro generate small, acapsular colonies. Acapsular, invasive phenotypes may thus not induce antibody defences, which might account for the lack of vaccine efficacy.

Choi et al. [31] suggest another mechanism for the lack of vaccine efficacy against serotype 3. They observed that, for the majority of serotypes, capsular polysaccharide (CPS) production proceeds along the wzy-dependent pathway, whilst serotype 3 uses a synthase-dependent pathway. This synthase-dependent pathway produces polysaccharide that is not covalently-linked to the peptidoglycan in the bacterial cell wall, but is instead bound on phosphatidylglycerol or the synthase on the membrane, and can be released by dissociation from the phosphatidylglycerol or ejection from the synthase. The authors compared CPS release in vitro and in vivo from various serotypes, and then determined whether the amount released was sufficient to inhibit antibody-dependent bacterial killing and protection against serotype 3. They found that cultured serotype 3 released approximately 60 μg/10^7^ CFUs compared to 0.4 to 10 μg/10^7^ CFUs for serotypes 1, 4, 6B, and 14. They also found that in vitro release was 31.2 μg/10^7^ CFUs compared with <0.1 μg/10^7^ CFUs for serotype 4 and 0.8 μg/10^7^ CFUs for serotype 5. The addition of 12 ng of purified CPS was sufficient for inhibition of killing in an opsonophagocytic killing assay from 90–100% to 50%. In order to determine capacity of released CPS to inhibit protection by passive transfer of anti-capsule antibodies in mice, the minimum amount of capsule-specific rabbit serum to provide ≥80% protection was administered intraperitoneally 24 h prior to challenge with 1000 CFU of serotype 3. In mice that were passively immunized with serum containing the relevant anti-capsule antibodies, the survival rate was reduced to ≤20% in the presence of as little as 0.03 μg of type 3 CPS. They concluded that “the failure of the serotype 3 conjugate in PCV13 may be a direct consequence of the CPS release by the organism”.

Nurkka et al. [32] conducted a randomised double-blind study to test the efficacy of PCV11-PD against AOM in infants in Finland. 154 infants received either three doses of PCV11-PD at 2, 4, and 6 months with a booster dose at 12–15 months; three doses of PCV11-PD at 2, 4 and 6 months with a single booster dose of PPV23 at 12–15 months; or three doses of hepatitis B vaccine at 2, 4 and 6 months with a single “booster” dose of PCV11-PD at 12–15 months. Blood samples were taken at 7 months and immediately before, and 28 days after, the final booster dose, and serotype-specific IgG concentrations measured using ELISA. They found that IgG levels against serotype 3 were significantly higher for the groups receiving PCV11-PD than the control (hepatitis B vaccine) group following the primary course (at 7 months), but there was no significant difference between the groups five months later (immediately before the booster). There was also no significant difference between the groups 28 days after the booster dose, though all three groups showed an elevated antibody concentration in response to the booster dose. 

In a review of hyporesponsiveness after vaccination with polysaccharide or glycoconjugate vaccines, Poolman and Borrow [33] concluded that the study by Nurkka et al. [32] showed evidence of hyporesponsiveness to the serotype 3 component of PCV11-PD. This was compared to a number of studies showing a similar result for PCV13, where a booster dose did not elicit an increased antibody response.

## 5. Luminex Assay Reproducibility

Pneumococcal serotype-specific antibody assays are covered by the UK National External Quality Assessment Service for Immunology, Immunochemistry and Allergy (UK NEQAS IIA). Multiple laboratories in the UK and overseas test aliquots from two samples distributed by the UK NEQAS IIA bi-monthly and the results from testing these distributions are analysed and compared by the service. Further details, including detailed descriptions of the analysis performed, are on the UK NEQAS IIA website [34].

**Figure 1:**
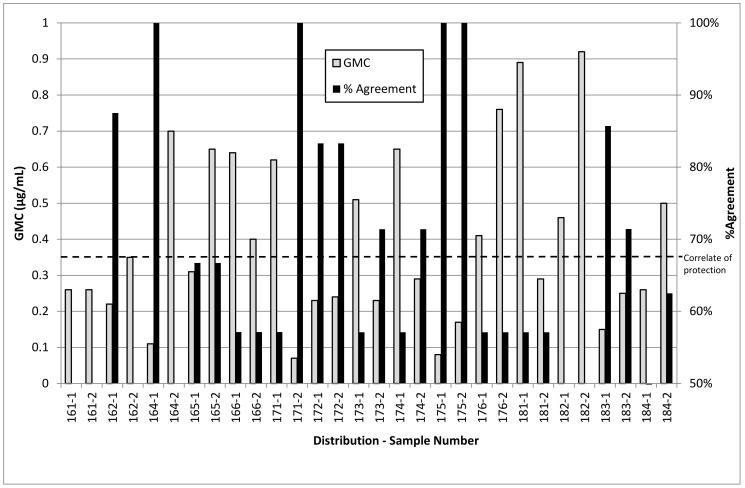
Chart showing mean serotype 3 specific IgG concentration compared to percentage agreement between labs for the UK National External Quality Assessment Service for Immunology, Immunochemistry and Allergy (UK NEQAS IIA) pneumoccocal antibody serotype-specific immunoassay distributions between 2016–2018. Positive results are those assigned a concentration greater than the correlate of protection (0.35 μg/mL) by that laboratory and negative results are those equal to or lower than the correlate of protection. Percentage of agreement equals the percentage of laboratories assigning the same result, with 50% being the lowest possible.

Figure 1 shows a summary of results submitted by the ten participating laboratories comparing the mean antibody concentration for serotype 3 with the percentage agreement in assigned results. Between six to eight laboratories returned results for any single sample. 

Agreement between laboratories for serotype 3 is low, and frequently 50% (i.e., no higher than chance), whilst the other 11 serotypes generally exhibited higher rates of agreement of ~90% compared with ~65% for serotype 3. Greater agreement for serotype 3 is found between samples of low mean concentration that would be expected to give unambiguously negative results. Poorer agreement is found between samples of higher concentration, even when these would be expected to give unambiguously positive results. This pattern is not seen with other serotypes—agreement is lower when samples had mean concentrations around the correlate of protection, as would be expected, but higher mean concentrations gave positive results with high agreement. This suggests that laboratory-to-laboratory reproducibility for the serotype 3 component of this assay is poor, and that results for this component may not be reliable.

## 6. Discussion

There is substantial evidence for the lack of efficacy of the serotype 3 component of pneumococcal vaccines [8,9,10,11,12,13,14,15,16,17,18,19,20]. This communication has detailed the results of ten studies from seven countries, all of which show negative or limited efficacy of PCV13 against *S. pneumoniae* serotype 3 infection or carriage. Other pneumococcal vaccines (conjugate or polysaccharide) have also shown limited or no efficacy against serotype 3 [21,22].

A number of studies have shown evidence of hyporesponsiveness against serotype 3 following vaccination with conjugate vaccines [23,32,33], suggesting that the development of lasting immunity to this serotype requires further study.

The work of Andrews et al. [12] has suggested that the current correlate of protection used for serotype 3 is more than eight-fold too low, thus even when relatively high antibody levels are produced, this may be insufficient to provide protection. Work to investigate the capsule of serotype 3 suggests that the unusually dense and thick capsule produced by this serotype may, through various mechanisms, provide a means to elude immune system protection. In addition, the ability of the organism to release free CPS may provide a further means to inhibit killing by antibodies.

The current multiplexed assay in use for the majority of clinical services to measure serotype-specific antibody levels in the UK demonstrates issues regarding the reproducibility of results, especially at higher concentrations of antibody. Were the correlate of protection to be increased according to the work of Andrews et al. [12], this issue is likely to become more serious due to the very poor agreement demonstrated at higher concentrations.

These factors combine to make the clinical measurement of serotype 3 specific antibodies of limited utility. It is not recommended that measurement of serotype-specific antibodies be used as part of the diagnosis of infection [6], so most measurements will be used to assess the efficacy of vaccine treatment, or to assess the ability of an individual to mount a specific antibody response. Controversy regarding the correct correlate of protection value [12] means that a “protective” result is likely incorrect unless the measured concentration is high, and poor inter-laboratory reproducibility raises questions about the reliability of any result. Due to the apparent rapidly waning serotype 3 antibody response following vaccination [12,23,24], and the possible hypo-responsiveness to repeat vaccinations [23,32,33], a negative antibody measurement gives no information about a vaccinated patient’s intrinsic ability to mount an antibody response, and thus this measurement is not useful in the diagnosis of immunological conditions. 

In order to address the issue of poor assay reproducibility, work would be required to measure the extent of the issue, determine the underlying causes, and provide solutions to these. Implementing these fixes would then require extensive revalidation of the assay. This work would require a substantial investment of money and time to undertake, and so serious thought must be given as to the usefulness of doing so, and the likely benefits to be gained. It is the opinion of the authors that, in the absence of a complete understanding of the immune response to serotype 3, the measurement of serotype 3 specific antibodies is of such limited clinical relevance that such work would be of little value at the current time. Instead, we suggest that consideration should now be given to removing the serotype 3 component of the serotype-specific immunoassay as part of a clinical service, and that much further work is required to understand the immune response to serotype 3 in order that incorporation into future vaccine formulations might be improved.
